# The influence of the increasing use of assisted reproduction technologies on the recent growth in fertility in Czechia

**DOI:** 10.1038/s41598-023-37071-7

**Published:** 2023-07-05

**Authors:** Jiřina Kocourková, Anna Šťastná, Boris Burcin

**Affiliations:** Department of Demography and Geodemography, Faculty of Science, Charles University, Prague, Czechia

**Keywords:** Public health, Health policy, Genetic services, Infertility

## Abstract

This study aims to enhance the understanding of how the increasing use of assisted reproductive technologies (ART) has contributed to the increase in the total fertility rate (TFR) and to further delaying childbearing. Moreover, it addresses the gap in the methodology concerning the quantification of the effect of ART on fertility postponement. Czechia is one of few countries that are able to serve for the study of the demographic impacts of ART. ART and non-ART fertility rates were calculated using unique data on all children born in Czechia. Excluding mothers who received cross-border reproductive care, the proportion of ART live births in Czechia has not exceeded 4%. However, without ART the TFR would have stood at just 1.65 instead of 1.71 in 2020. ART significantly contributed to a reduction in childlessness and to the increase in fertility rates at ages over 35. Applying the decomposition method, the contribution of the use of ART to delaying childbearing between 2013 and 2020 was 4%. The findings have important policy implications. ART has the potential to support fertility recovery in the context of delayed childbearing. The findings served to alleviate concerns about the contribution of ART to the further undesired delay of childbearing.

## Introduction

Czechia has had low fertility rates (TFR of below or around 1.5 children per woman) since the 1990s^[1]^. In recent years, however, fertility has been increasing and the TFR reached 1.71 children per woman in 2020. As a result, Czechia currently has one of the highest fertility rates in the EU together with France (1.82) and Romania (1.78)^[2]^. In addition, assisted reproductive technology (ART), e.g. in vitro fertilisation (IVF) has become an important component of demographic reproduction over the last two decades in line with its increased use worldwide^[3]^. Czechia is one of a number of countries that have consistently maintained a high percentage of ART births since 2005 to the present (data from 2018): Denmark (5.7%), Iceland (5.6%), Czechia (5.5%) and Belgium (5.0%)^[3–5]^. This raises the question as to the extent to which the growth in the TFR in Czechia was driven by the increasing use of ART.

To date, research has focused mainly on the medical aspects of ART, e.g. the risk of adverse birth outcomes^[6–8]^, whilst demographic studies are rare when one considers the increasing importance of ART for demographic reproduction. The more intensive use of ART is associated with a trend towards delayed childbearing and low fertility^[9,10]^; thus, it has led to governments enhancing the availability of ART as a potential tool to counteract the effect of declining fecundity with age^[11]^ and the integration of ART with policies aimed at supporting childbearing^[12–14]^. Conversely, some remain sceptical concerning the effect of ART on fertility rates^[15,16]^ emphasising the associated costs and side effects^[17]^. Moreover, concerns have been raised about the extent to which the increased use of ART acts to support further delays in childbearing by creating the illusion that fertility can be delayed until late reproductive ages^[18]^. In contrast, other studies argue that the availability of ART encourages couples to seek help sooner rather than later^[19,20]^. All these factors are being considered in the debate on whether to increase the availability of ART by raising the age limit for access to, and the reimbursement of the costs of, this method^[21]^.

Accordingly, the quantification of both the contribution of ART to the TFR and age-specific fertility rates and its impact on childbearing postponement is relevant in terms of the creation of evidence-based policies. However, very few studies have been conducted on the measurement of the demographic implications of ART use. Concerning most European countries, the lack of the relevant data is the main reason for the paucity of such studies. Recent Scandinavian studies based on population registers are an exception; however, they have tended to concentrate on the research of the socio-demographic profiles of parents who have children using ART rather than the demographic implications^[22]^. A cohort analysis of Danish data^[18]^ indicated that ART can contribute to fertility; however, it is somewhat outdated.

European countries suffer from a research gap concerning the importance of ART for the recovery of fertility at older reproductive ages. Although a number of non-European studies are available, they reflect different contexts and ART legislation environments. A recent US study acknowledged the increasing impact of ART on fertility trends^[23]^. Similarly, an Australian study revealed that the recent increase in fertility rates at advanced reproductive ages is largely driven by the increasing use of ART^[9]^. Thus, concerning most European countries, there remains a lack of studies that assess the potential role of ART in terms of supporting fertility recovery in the context of delayed childbearing. Despite the existence of the European IVF monitoring of data on ART treatment cycles collected by ESHRE, data on births following ART is often incomplete due to its having no link to national birth registers.

**Figure 1:**
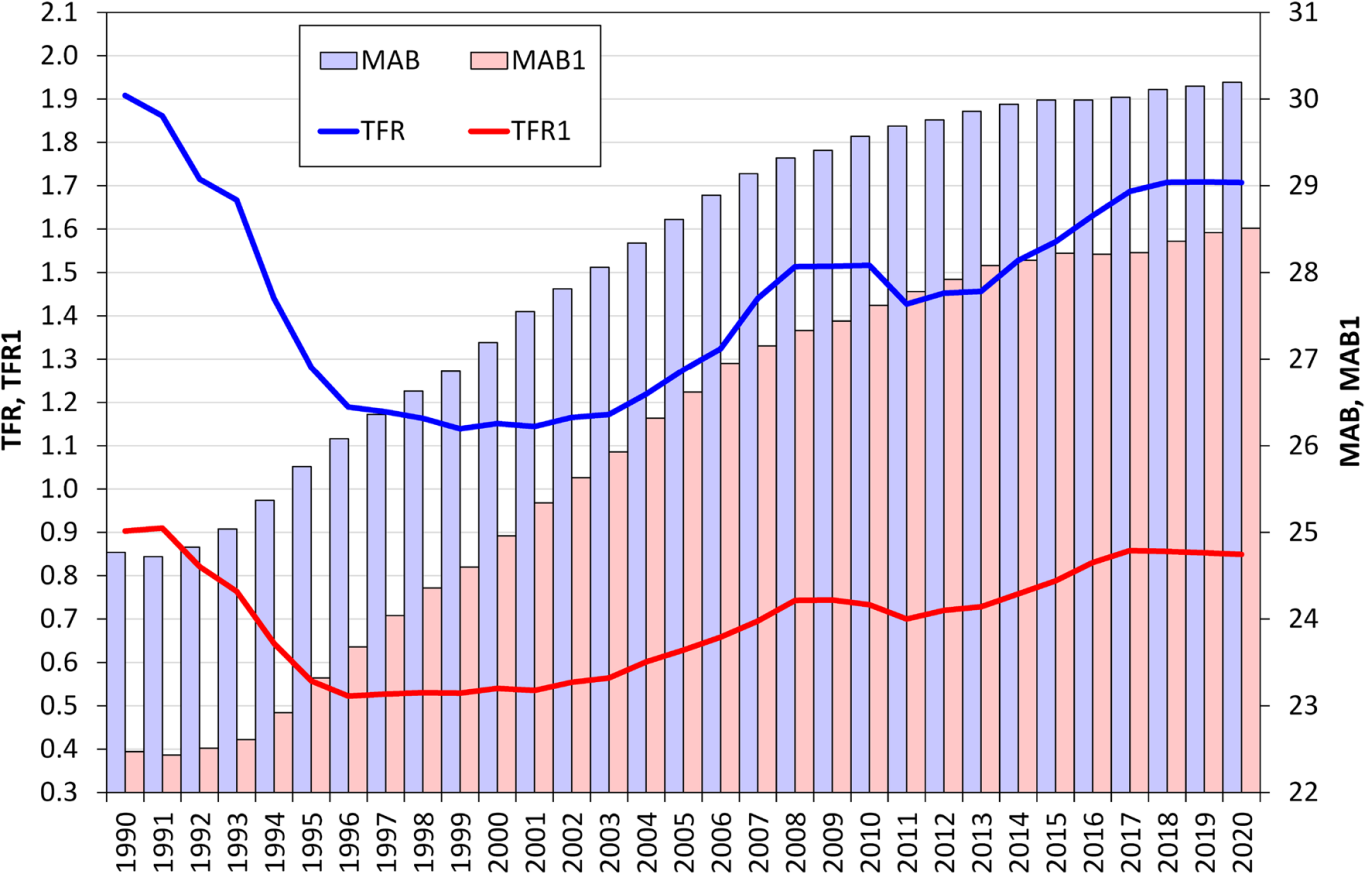
Total fertility rate (TFR), total fertility rate of parity 1 (TFR1), mean age of women at birth (MAB) and mean age of women at first birth (MAB1), Czechia, 1990–2020. Data source: CZSO 2023.

Czechia is unique in terms of providing an opportunity to study the demographic impacts of ART in terms of its being one of the few European countries to record a significant increase in ART use immediately following the introduction of ART legislation and registration in 1997^[5]^. Moreover, Czechia registered one of the most dramatic shifts to the late childbearing pattern, as evidenced by a sharp fall in the TFR from 1.90 in 1990 to the lowest level of 1.13 in 1999 accompanied by an extraordinarily rapid increase in the mean age of women at first childbirth from 22.5 in 1990 to 24.6 in 1999^[24]^ (Fig. 1). Czechia has, since this time, consistently topped the list of countries in terms of ART use^[3]^ and it has been argued that the increasing use of ART was one of the factors that contributed to Czechia moving from its lowest TFR at the beginning of the 2000s to 1.5 in 2008^[5]^. From 2013, the increase in the TFR accelerated remarkably in Czechia. While in 2013 the TFR stood at 1.53, by 2020 it had reached 1.71; thus, Czechia now has one of the highest TFRs in Europe. The increase in the mean age of women at first birth slowed down after 2013 to reach 28.5 in 2020 and stabilised at a level of around the EU average, in contrast to most western European countries that registered higher mean ages of women at childbirth^[25]^. Moreover, Czechia still has one of the highest rates of ART use^[3]^ and has become a major target country for cross-border reproductive care^[26]^. Therefore, the research question posed in this study concerns the extent to which the relatively high use of ART has contributed to the country’s high TFR. Despite several studies having been conducted on the situation in Czechia^[5,27]^, it was not possible to provide a detailed analysis due to the non-availability of individual data on ART use prior to 2013. In addition, it was not possible to examine the effect of ART use on fertility until women who received cross-border reproductive treatment could be excluded from the data.

The key benefit of this study concerns the use of a unique database that was created by linking anonymised individual data on each live born child from the vital statistics of the Czech Statistical Office to data from the National Register of Assisted Reproduction administered by the Institute of Health Information and Statistics of the Czech Republic. The aims of this study are to (1) quantify the contribution of ART to the increase in the TFR and age-specific fertility rates, and (2) measure the impact of ART on birth timing applying the decomposition method. We aimed to address the extent to which the increase in ART use has contributed to further delaying childbearing and its potential support for the postponement of childbearing. The contribution of this study concerns the enhancement of our understanding of the effect of ART in the context of an increasing TFR accompanied by a slowdown in fertility postponement. Secondly, we address the gap in the methodology concerning the assessment of the effect of ART on fertility postponement.

## Legislation on ART use in Czechia

ART has been easily available in Czechia since the initial legislation was introduced on the reimbursement of ART costs by the statutory health insurance system in 1997^[5]^; women became entitled to the reimbursement of the costs of up to three cycles of ART treatment up to the age of 39. In April 2012, an age limit of 50 years was introduced for access to ART, while the age limit of 39 for entitlement to costs reimbursement remained unchanged, and the number of reimbursed cycles was increased to four provided that only one embryo was transferred during each of the first two cycles^[21]^. The number of ART clinics in Czechia compared to the country’s population is one of the highest in Europe^[28]^. All ART treatments, e.g. in vitro fertilisation (IVF), intracytoplasmic sperm injection (ICSI), frozen embryo transfer (FET), egg donation, oocyte and embryo receipt and sperm donation are permitted. However, ART is not fully available for all women in Czechia since single women and same-sex partners do not have direct access to ART. All women who apply for ART treatment must provide the written consent of their male partner; however, this does not mean that they have to be married or cohabitate. Moreover, the statutory health insurance system does not cover all the costs. One cycle usually costs EUR 2400–3000^[26]^, up to one-third of which is usually paid by the patient since some modern drugs and treatments, e.g. ICSI, AH (assisted hatching) and FET, are not covered. Czechia has also become a major target country for cross-border reproductive care, due mainly to its liberal legislation, the availability of suitable donors (and the assurance of anonymity) and the high quality and safety of the treatment provided. The proportion of IVF cycles received by non-Czech women accounts for 26% of the total, 41% regarding FET cycles, and as high as 86% concerning oocyte receipt cycles^[26]^.

## Data and methods

Data on ART is recorded in the National Register of Assisted Reproduction (NRAR) administered by the Institute of Health Information and Statistics of the Czech Republic (IHIS). The reporting of data on ART cycles has been legally required for all reproductive centres/clinics since 2006. However, up to 2012 this information was not sufficiently complete to allow for the identification of Czech ART live births. Therefore, we considered data from 2013 up to the last available year, 2020. It was possible to link the individual data on live births from the vital statistics of the Czech Statistical Office to the NRAR database via the women´s birth (ID) number so as to identify those live births that followed successful ART treatment. The linkage was implemented by the IHIS and the dataset was further analysed in the fully anonymised form. Thus, our data was not distorted by cross-border reproductive care, which has no relevance to fertility in Czechia.

Both the vital statistics and the NRAR contain precise dates (day/month/year), which make it possible to define ART births as live births that followed an embryo transfer a maximum of 40 weeks before the due date. In addition, the gestational age at birth was verified. Thus, we covered all those ART cycles that aimed to achieve a pregnancy and in which embryo transfer took place. The potential underestimation of the number of ART births as a result of having no data on women who had ART treatment abroad but who are included in the Czech vital statistics of new-borns is negligible since receiving ART abroad is rare for Czech women considering that Czechia is a major target country for cross-border reproductive care. Nevertheless, the albeit low overestimation of the number of ART births due to the possibility that some women became pregnant via sexual intercourse following ART cannot be excluded^[29,30]^.

The initial aim was to quantify the impact of the use of ART on the TFR. The standard fertility indicators were re-defined in terms of the use/non-use of ART. The number of live births conceived without the use of ART was obtained by subtracting the number of ART live births from the total number of live births for each year and the mother’s age. Accordingly, the fertility model can be described as follows:

1 $$TFR={TFR}^{nonART}+{TFR}^{ART}={\sum _{x}}{f}_{x}^{nonART}+{\sum _{x}}{f}_{x}^{ART}={\sum _{x}}\frac{{B}_{x}^{nonART}}{{P}_{x}}+{\sum _{x}}\frac{{B}_{x}^{ART}}{{P}_{x}}$$

where B_x_ represents the number of live births to women of age x and conceived without (nonART) or with (ART) assisted reproductive technologies. P_x_ represents the number of women at age x and f_x_ is the age specific fertility rate for ART or nonART fertility. In addition, the fertility model can be specified by the parity.

The second aim was to quantify the effect of the use of ART on birth timing.

The mean age of women at birth (MAB) can be specified as:

2 $${MAB}^{ART}=\frac{{\sum\limits _{x}}{f}_{x}^{ART}*x}{{\sum\limits _{x}}{f}_{x}^{ART}}$$

for ART fertility and

3 $${MAB}^{nonART}=\frac{{\sum\limits _{x}}{f}_{x}^{nonART}*x}{{\sum\limits _{x}}{f}_{x}^{nonART}}$$

for fertility without ATR treatment.

We presupposed that the increasing use of ART contributes to the postponement of childbearing. Thus, the decomposition method was applied to distinguish between the effect solely of childbearing postponement and the effect of ART use. Since the mean age of women at birth (MAB) has increased, it is possible to quantify how much of this increase between 2013 and 2020 was due to the increasing use of ART. The MAB over time *t* can be expressed as a weighted average where *nonART TFR* and *ART TFR* are taken as weightings. We applied a formula that was developed for the decomposition of the change in the *MAB* according to the *TFR* by birth order, i.e. where *i* represents the birth order (*i* = 1, 2, 3, etc)^[31,32]^. We took *ART* and *nonART* fertility as the variable *i* instead of the birth order: *i* = ART; nonART

4 $$MAB(t)=\frac{{\sum\limits _{i}}{MAB}^{i}\left(t\right)\times {TFR}^{i}(t)}{TFR(t)}$$

The decomposed change in the *MAB* between years *t* and *t* + *h* can then be expressed as follows:

5 $$\Delta {\overline{MAB} }_{(t,t+h)}=\left[\left(\frac{\frac{{TFR}^{nonART}(t+h)}{TFR (t+h)}+\frac{{TFR}^{nonART}(t)}{TFR (t)}}{2}\right)\times \left({MAB}^{nonART}\left(t+h\right)-{MAB}^{nonART}\left(t\right)\right)\right]+\left[\left(\frac{\frac{{TFR}^{ART}(t+h)}{TFR (t+h)}+\frac{{TFR}^{ART}(t)}{TFR (t)}}{2}\right)\times \left({MAB}^{ART}\left(t+h\right)-{MAB}^{ART}\left(t\right)\right)\right]+\sum_{i}\frac{{MAB}^{i}\left(t+h\right)+{MAB}^{i}\left(t\right)}{2}\times \left(\frac{{TFR}^{i}\left(t+h\right)}{TFR(t+h)}-\frac{{TFR}^{i}\left(t\right)}{TFR(t)}\right)=\Delta {MAB}_{timing}^{nonART}\left(t, t+h\right)+\Delta {MAB}_{timing}^{ART}\left(t, t+h\right)+\Delta {MAB}_{structure}\left(t, t+h\right)$$

The first two components represent the *effect of nonART timing* and the *effect of ART timing*, i.e. changes in the *MAB* due to the real increase in the mean age of mothers, and the third component represents the *effect of structure,* i.e. changes in the *MAB* due to changes in the fertility structure according to *ART* and *nonART*.

## Results

### Impact of the use of ART on the fertility level

The numbers of all live births and live births following ART exhibited the same trend—a consistent increase between 2013 and 2018 followed by a slight decrease in 2019–2020 (Table 1). The highest number of ART live births was registered in 2018, i.e. 3.8% of all live births. By 2020 the proportion of ART live births had stabilised and did not exceed 4%. Given that the proportion of multiple births was significantly higher in 2013 than in 2020, when the promotion of single embryo transfer in the reimbursement policy of Czechia contributed significantly to the decline, we focus on deliveries in which at least 1 live child was born (Table 1). The results show the even more dynamic increase in the number and proportion of ART deliveries compared to ART live births. While the number of ART live births increased by 15% between 2013 and 2020 and the proportion of ART live births of total live births increased by 0.38 percentage points, there was a 26% increase in the number of ART deliveries, and the proportion of ART deliveries of all deliveries increased by 0.65 percentage points. With respect to the number of deliveries with at least one live birth, the increasing impact of ART on reproduction is more pronounced than when one considers the number of live births. Indeed, the number of ART live births reflects the higher proportion of multiple pregnancies following ART treatment, particularly at the beginning of the study period.

**Table 1:** Deliveries with live birth, live births, the TFR and MAB by the use and non-use of ART, Czechia 2013–2020.

	2013	2014	2015	2016	2017	2018	2019	2020
Total number of deliveries with live birth	104,956	108,184	109,144	111,001	112,812	112,539	110,779	108,745
Number of deliveries with live birth following ART	3168	3629	3607	3742	3995	4084	4083	3985
Deliveries with live birth following ART (%)	3.02	3.35	3.30	3.37	3.54	3.63	3.69	3.66
Total number of live births	106,751	109,860	110,764	112,663	114,405	114,036	112,231	110,200
Number of live births following ART	3604	4032	3907	4035	4277	4309	4286	4140
Live births following ART (%)	3.38	3.67	3.53	3.58	3.74	3.78	3.82	3.76
Total fertility rate (TFR)	1.456	1.528	1.570	1.630	1.687	1.708	1.709	1.707
Total fertility rate following ART (TFR^ART^)	0.045	0.052	0.051	0.053	0.057	0.058	0.059	0.058
Total fertility rate without ART (TFR^nonART^)	1.411	1.476	1.519	1.577	1.629	1.650	1.650	1.649
TFR^ART^/TFR (%)	3.11	3.38	3.23	3.26	3.39	3.42	3.46	3.39
Total mean age of women at birth (MAB)	29.86	29.94	29.99	29.99	30.02	30.11	30.15	30.19
Mean age of women at birth following ART (MAB^ART^)	33.67	33.85	34.04	34.29	34.40	34.54	34.51	34.58
Mean age of women at birth without ART (MAB^nonART^)	29.74	29.80	29.86	29.84	29.87	29.95	30.00	30.04
Difference between MAB^ART^ and MAB^nonART^	3.93	4.05	4.18	4.45	4.54	4.58	4.51	4.54

Data source: CZSO 2023, IHIS 2022.

Table 1 also shows the significant increase in the TFR between 2013 and 2018 from 1.46 to 1.71 and from 0.045 to 0.058 for the ART TFR followed by the stabilisation of these values up to 2020. As a result, the relative impact of ART on the TFR did not increase significantly—from 3.1% in 2013 to 3.4% in 2020. Nevertheless, the increasing use of ART contributed to the increase in the overall TFR; the TFR in 2020 without ART would have reached just 1.65 instead of 1.71 with ART.

The TFR was further analysed by parity separately for the nonART and ART fertility rates in order to quantify which groups of women use ART the most, childless women or those with at least one child (Table 2). First parity fertility was confirmed as the driver of the increase in the ART TFR, with the ART TFR1 registering a 38% increase between 2013 and 2020 from 0.028 to 0.038. As a result, the share of the ART TFR1 of the ART TFR increased from 61 to 66%, while the share of the nonART TFR1 remained unchanged and represented around 50% of the nonART TFR for the whole period. Thus, ART is becoming increasingly important, especially for childless women seeking to fulfil their maternal aspirations.

**Table 2:** Total fertility rates (TFR) by parity and the mean age of women at first childbirth (MAB1), nonART and ART, Czechia 2013–2020.

	TFR	TFR1	TFR2	TFR3+	Share TFR1 (%)	MAB1
nonART						
2013	1.411	0.700	0.517	0.194	49.6	27.89
2014	1.476	0.725	0.542	0.209	49.1	27.92
2015	1.519	0.755	0.555	0.209	49.7	28.01
2016	1.577	0.795	0.566	0.216	50.4	27.99
2017	1.629	0.821	0.583	0.226	50.4	27.99
2018	1.650	0.817	0.602	0.230	49.5	28.11
2019	1.650	0.814	0.607	0.229	49.3	28.22
2020	1.650	0.811	0.606	0.233	49.2	28.26
Change 2013–2020 (%; years)	16.9	15.9	17.2	20.0		0.36
ART						
2013	0.045	0.028	0.014	0.003	61.1	32.84
2014	0.052	0.032	0.016	0.004	62.2	33.05
2015	0.051	0.032	0.015	0.003	64.0	33.26
2016	0.053	0.034	0.016	0.004	64.0	33.49
2017	0.057	0.037	0.017	0.004	64.4	33.65
2018	0.058	0.038	0.016	0.004	65.8	33.73
2019	0.059	0.039	0.017	0.004	65.4	33.64
2020	0.058	0.038	0.016	0.003	66.1	33.78
Change 2013–2020 (%; years)	27.6	38.1	14.2	–1.6		0.93

Data source: IHIS 2022.

**Figure 2:**
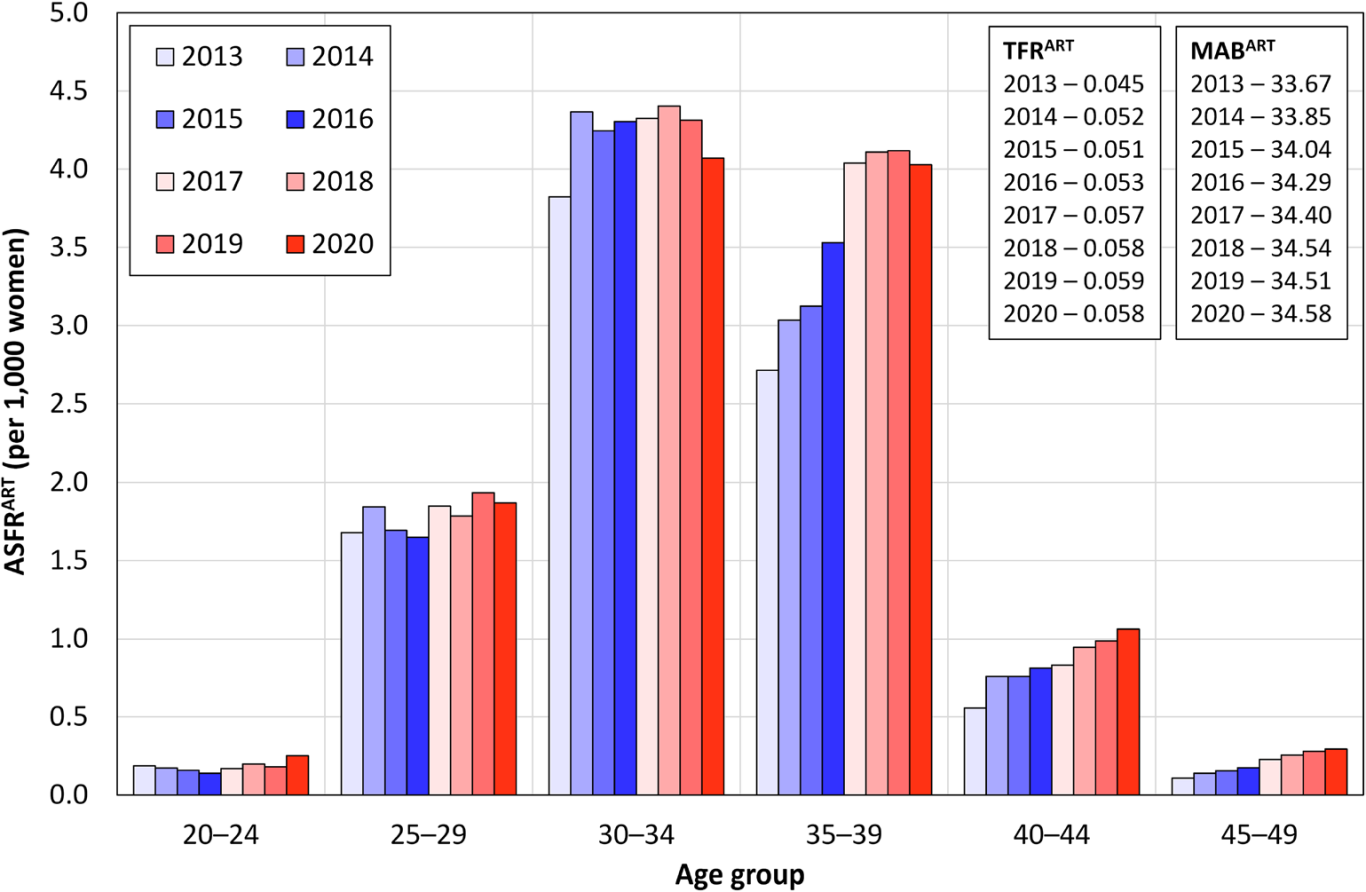
Age-specific fertility rates following ART by age group, Czechia 2013–2020. Data source: IHIS 2022.

Figure 2 shows that the highest ART fertility rates concerned the 30–34 age group during the period under study. However, the most dynamic development of ART fertility was evident regarding the 35–39 age group, in which the highest increase in the ART fertility rates was concentrated. This served to confirm the significant role of ART in terms of compensation for delaying childbearing to older ages. In addition, the impact of Czech ART legislation is reflected in this increase, i.e. the entitlement to cost reimbursement ends at age 39 years and women tend to time ART treatment so as to take advantage of this opportunity. As a result, the clear dominance of the 30–34 age group in 2013 had waned by 2020 so that the 35–39 age group matched the 30–34 age group in terms of ART fertility rates. Nevertheless, the relatively young age profile of ART fertility continued to dominate during the period under study with ART fertility rates for the 25–29 age group remaining significantly higher than for the 40+ age group despite the continuous increase evident concerning the 40–44 age group.

Aimed at assessing the age-specific contribution of ART use to the overall TFR, the sum of the age-specific fertility rates was calculated for nonART and ART use (Table 3). Although the sum of age-specific fertility rates for both nonART and ART evinced a distinct increase regarding women aged 35 and over, the increase in ART fertility was more profound and became the key driver of fertility increases at more advanced ages. While the share of the 35+ age group of total nonART fertility increased from just 16% in 2013 to 18% in 2020, the share of the 35+ age group of total ART fertility increased significantly from 37% in 2013 to 46% in 2020. As a result, the share of ART fertility remained unchanged and remained low for the 30–34 age group (less than 4% of overall fertility rates), while it increased for the 35–39 age group (almost 8% of overall fertility rates). This share increased most for those aged 40+ (from 9% in 2013 to 12% in 2020).

**Table 3:** Sum of age-specific fertility rates per age group (per 1,000 women), nonART and ART, Czechia 2013–2020.

	nonART					ART					ART/(nonART + ART) (%)			
	–29	30–34	35–39	40+	35+ (%)	–29	30–34	35–39	40+	35+ (%)	30–34	35–39	40+	35+
2013	713.7	477.4	187.2	32.3	15.6	9.3	19.1	13.6	3.3	37.3	3.8	6.8	9.3	7.1
2014	736.6	503.9	203.0	32.5	16.0	10.1	21.8	15.2	4.5	38.1	4.2	7.0	12.1	7.7
2015	755.1	512.0	214.8	37.4	16.6	9.3	21.2	15.6	4.6	39.8	4.0	6.8	10.9	7.4
2016	786.5	525.7	225.5	39.2	16.8	9.0	21.5	17.6	4.9	42.6	3.9	7.3	11.2	7.9
2017	813.0	539.1	235.2	42.2	17.0	10.1	21.6	20.2	5.3	44.6	3.9	7.9	11.2	8.4
2018	819.0	541.6	242.8	46.5	17.5	9.9	22.0	20.5	6.0	45.4	3.9	7.8	11.4	8.4
2019	820.7	536.3	245.4	47.6	17.8	10.6	21.6	20.6	6.3	45.6	3.9	7.7	11.7	8.4
2020	820.7	532.2	247.8	48.8	18.0	10.6	20.4	20.1	6.8	46.5	3.7	7.5	12.2	8.3
Change 2013–2020 (%)	15.0	11.5	32.4	50.9		13.7	6.5	48.4	103.5					

Data source: IHIS 2022.

**Figure 3:**
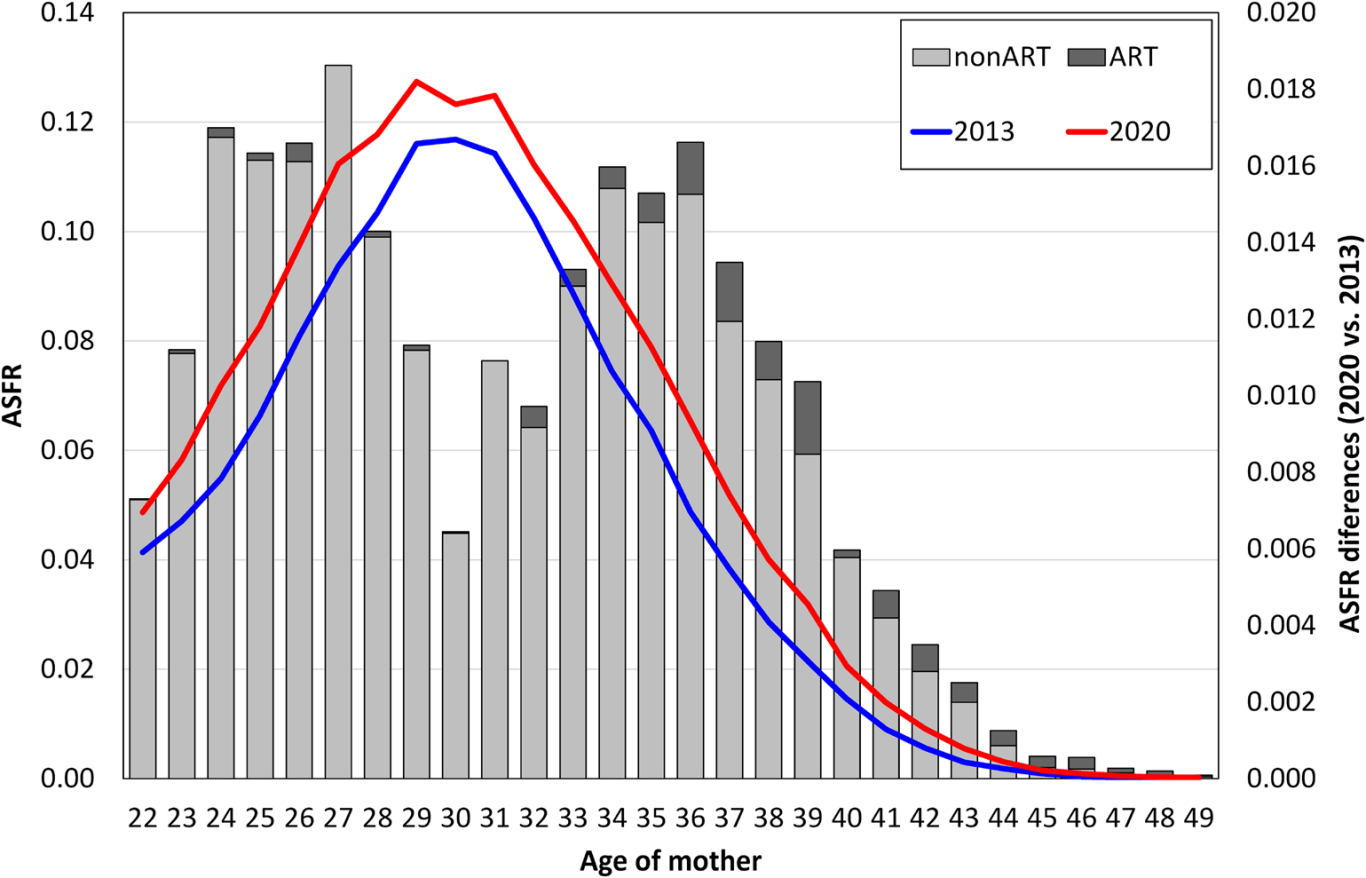
Age-specific fertility rates (ASFR) in 2013 and 2020, differences in the ASFR between 2013 and 2020 for nonART and ART fertility. Data source: CZSO 2023, IHIS 2022.

We subsequently analysed in greater detail the extent to which the change in the overall TFR between 2013 and 2020 was reflected in the age fertility profile. Figure 3 provides a summary of the role that changes in the age-specific ART and nonART fertility rates played in determining the observed increase in the overall TFR. Although an increase in fertility rates was determined across all ages, the contributions of ART and nonART fertility differed. Interestingly, the changes exhibited a two-peak curve. The increase in the age-specific fertility rates of women in their mid-30s and late 30s can be understood to be the result of the postponement process; however, the increase in the age-specific fertility rates of women at younger ages concentrated at around the mid-20s suggests the cessation of fertility postponement. The increase in the overall TFR was largely balanced between younger and older ages, which resulted in a flattening and a widening of the fertility curve. Figure 3 also shows that the increase in fertility at age below 30 was due mainly to the increase in natural (nonART) fertility, while the increase in ART fertility played a significant role at ages over 30. The lower the increase in nonART fertility at advanced ages, the higher the compensation via an increase in ART fertility. ART fertility rates at ages 36 to 39 accounted for the largest share of fertility recovery. Clearly, without ART, fertility recovery at more advanced ages would have been markedly lower due to the significantly reduced natural fertility of women at such ages.

### Impact of the use of ART on birth timing

The second aim was to quantify the effect of the use of ART on birth timing. The ART mean age at birth (MAB) was 4 years higher than the nonART MAB in 2013, and the difference had increased to 4.5 years by 2020 (Table 1). The ART MAB increased by 0.9 years from 2013 to 34.6 years in 2020, while the nonART MAB increased by only 0.3 years from 2013 to reach 30 years in 2020. The increase in ART use was, partly, a consequence of the fertility postponement process and, partly, a contributing factor for fertility postponement since women that gave birth following ART had become significantly older.

**Figure 4:**
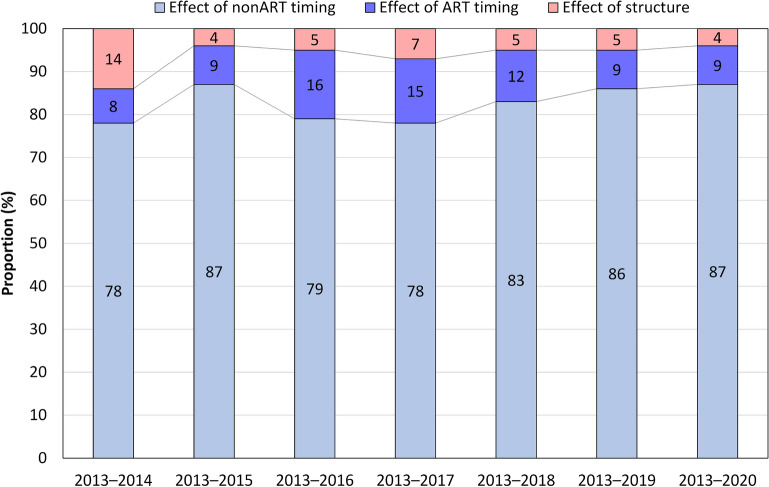
Decomposition of the increase in the MAB between 2013 and 2020 into the effect of fertility postponement (nonART and ART fertility timing) and the effect of the increase in the use of ART (structure) (in %). Data source: IHIS 2022.

Applying the decomposition method, it was possible to determine how much of the increase in the MAB can be attributed to changes in the timing of fertility (the effect of pure fertility postponement) and how much can be attributed to changes in the fertility structure dependent on the use or non-use of ART (the effect of the increased use of ART). Furthermore, the effects of nonART and ART fertility timing were separated so as to allow for a better understanding of the contribution of ART timing (Fig. 4). The results show that 14% of the increase in the MAB between 2013 and 2014 can be attributed to the increase in ART use. However, by 2020 this effect had decreased to just 4% suggesting that the availability of infertility treatment has significantly reduced its potential negative influence in terms of inducing couples to delay childbearing until late reproductive ages. The impact of ART timing was greatest between 2013 and 2016, when 16% of the increase in the MAB can be attributed to the transfer of the timing of ART to older ages. Moreover, it had decreased by 2020, which can be attributed to improvements in ART success rates. The decomposition analysis confirmed that the effect of nonART fertility postponement was the key driver in the increase in the MAB; moreover, its contribution increased from 78% in 2014 to 87% in 2020.

## Discussion

The aim of the research was to assess the extent to which the growth in the TFR since 2013 in Czechia has been driven by the increasing use of ART. Despite there being other relevant factors behind the continuous increase in the TFR in Czechia over the last decade, in particular economic growth that ensured stable conditions for young people to start a family and improvements in family policy^[25,33]^, the overall long-term fertility postponement process has resulted in the increased need for the use of ART.

Even though ART cannot compensate for all the fecundity lost by delaying attempts at conceiving^[34]^, our findings confirmed that the increase in the use of ART is relevant when assessing recent fertility trends. The impact of ART on the TFR in Czechia was significant; between 2013 and 2020 the contribution of ART to the TFR increased from 0.045 to 0.058, i.e. from 3.1 to 3.4% of the TFR. As a result, in 2020 the TFR without ART would have been just 1.65 instead of 1.71 with ART.

The contribution of ART to the TFR in Czechia in 2020 (0.058) reached a value between that for the US in 2020 (0.023^[23]^) and for Australia in 2017 (0.088^[9]^). This shows the wide variation in the impact of ART on fertility, which reflects differences in the broader social context between the US, Europe and Australia. The US and Australia represent two extremes—from low support for ART in the US, where the use of ART is often expensive even with insurance coverage^[23]^), to generous and supportive funding with no restrictions based on age, parity or the number of cycles in Australia^[9]^. Czechia has adopted a typical European legislative approach in which a centralised health insurance system provides the reimbursement of a significant part of ART costs up to a certain female age limit (39 years in 2022) and for a limited number of cycles (3–4). Moreover, the high degree of accessibility of ART together with the high quality and safety of the treatment provided and relatively lower costs have established Czechia as an important target for cross-border reproductive care in the European context. However, potential still remains for increasing accessibility to ART for Czech women via the removal or age extension of the limits set for the reimbursement of ART costs by the health insurance system. Moreover, the debate on extending access to ART beyond heterosexual couples and the right of single women to apply for ART has recently begun to gain momentum^[35]^.

The demographic analysis of fertility by age and parity confirmed that first parity was the driver of the increasing impact of ART on the TFR between 2013 and 2020. Thus, ART has made a contribution to reducing childlessness rather than to increasing family size. The most dynamic increase in ART fertility rates was registered at ages of over 35, which confirmed the significant role of ART in the recovery of fertility at older reproductive ages.

The study also aimed to measure the impact of ART on birth timing via the assessment of the extent to which the increase in ART use has contributed to further delaying childbearing. Czechia is specific in terms of its increasing TFR accompanied by a slowdown in fertility postponement. The mean age of women at birth following ART increased by 0.9 years from 2013 to reach 34.6 years in 2020. Nevertheless, it was not confirmed that the use of ART serves to support the postponement of childbearing. The decomposition analysis results indicated that by 2020, the contribution of ART use to delaying childbearing had diminished to 4%. The increase in the MAB between 2013 and 2020 was mainly attributed to the ongoing nonART postponement of childbearing, accounting for 87% of the increase.

The data enabled us to define how many ART children were born in Czechia. The proportion of ART live births in Czechia in 2020 did not exceed 4%. Our results confirmed that the 5.5% of ART births recently reported for Czechia by Wyns^[3]^ was an overestimation due to the inclusion of births to women who received cross-border reproductive care in Czechia.

Interestingly, in 2013 the Czech ART TFR commenced at the level projected for the US ART TFR up to 2040 (0.048^[23]^). Thus, our findings confirmed that Czechia was in a unique position in terms of the relatively significant contribution of ART to the quantum of fertility as early as one decade ago (in a similarly way as in Denmark^[18]^). The impact of ART use on the TFR in Czechia must have increased significantly prior to 2013 when the TFR was below 1.5. The increasing use of ART helped Czechia to escape from the low fertility trap^[5]^. Interestingly, the availability of ART in Czechia before 2013 was increasing due mainly to the growing number of clinics, since no special policy intervention was recorded regarding ART legislation or costs reimbursement. In addition, prior to 2013, the postponement of childbirth was more pronounced, with the significant contribution of the conflict between family and work partly as a result of insufficient and inadequate developments in family policy and the economic recession^[25,36]^.

The use of ART continued to increase in Czechia from 2013 partly due to improvements in ART reimbursement conditions, i.e. an increase in reimbursed cycles from three to four in 2012. Despite a continuous increase in the ART TFR, the relative contribution of ART to the TFR did not increase significantly since the nonART TFR also increased significantly, reaching a peak of 1.65 in 2018. This coincided with family policy developments, the introduction of the 2017 Family Policy Concept and the inclusion of state support for ART in the Concept. It is evident that ART policy alone is unable to provide a solution to low fertility; rather, it should be included in broadly-conceived long-term policies^[5,17,19,21,23]^. Therefore, recent policy improvements based on the 2017 Family Policy Concept in Czechia^[25]^ may have acted to retard the increase in the contribution of ART use to the TFR.

Despite the results indicating the recent stabilisation of ART use in Czechia, an increase in demand for ART is likely in the near future, especially concerning women of older and advanced ages. This could lead to a further increase in the use of ART for the following reasons: firstly, changes in ART legislation aimed at expanding its accessibility. Indeed, in 2022 the extension of the age limit for the reimbursement of ART costs from age 39 to 40 was introduced in Czechia, the impact of which can be expected as early as in 2023 in connection with an increase in the contribution of ART to fertility rates at ages 40 and 41. Secondly, any increase in the potential of ART will depend on improvements in success rates and the emergence of alternative treatment options^[37]^. Thirdly, the Covid-19 pandemic-related uncertainty and the fear of an economic recession^[38,39]^ accompanied by a decrease in family support may result in further delays to childbearing, thus creating an increase in the potential use of ART. Public attitudes towards ART, i.e. the level of its social acceptance, may also play an important role. Szalma and Djundeva^[40]^ determined that the mean age of women at first birth has a statistically significant association with the acceptance of ART at the national level. Thus, the more women postpone the transition to motherhood in society, the more permissive attitudes to ART become. This may be due to the fact that women at later age are more likely to face fertility problems.

ART is only one of the treatment options that can be classified under the broader term of medically assisted reproduction (MAR). MAR for the treatment of infertility also includes more traditional treatments such ovulation induction, with or without intrauterine insemination (IUI)^[41]^. However, due to the lack of data it is not possible to properly assess the overall effect of MAR treatment on fertility in Czechia. Although the data indicates the additional impact of IUI on fertility in Czechia, i.e. around 0.9% of all live births annually in the period 2016–2018 (IHIS 2021), it is likely that this figure is underestimated since data on IUI is not validated and is based on incomplete evidence. The National Register of Assisted Reproduction does not track IUI or alternative treatment approaches other than ART. To assess in detail the overall effect of MAR on fertility levels, which is undoubtedly greater than the effect of ART alone, it would be necessary to expand the data to include evidence on these treatments and their outcomes.

In summary, the Czech TFR would be significantly lower without births conceived via ART, and childlessness would be more commonplace due to the low recovery of fertility at advanced reproductive ages. Interestingly, the findings also indicated the alleviation of concerns about the potential contribution of ART to the further undesired delay of childbearing. The findings have important implications for family policy. Based on experience in Czechia, it appears to be favourable to include ART policies in the overall policy mix aimed at assisting people to fulfil their reproductive plans. The importance of ART in terms of supporting fertility recovery in the context of delayed childbearing will continue to increase, as indicated by recent demographic trends in some European countries^[42]^.

When interpreting these results, it is important to note two limitations. Firstly, the slightly lower number of births in 2020 may have been influenced by the Covid-19 pandemic. However, ART clinics in Czechia were not closed until March 2020, which was reflected in a reduction in births 9 months later, i.e. in December 2020. Secondly, in some cases, it is possible that women would have had a baby later without the use of ART^[30]^. However, this would not have translated into the cross-sectional indicators that we observed. Moreover, the number of such births would have been negligible since ART is used mainly by older women concerning whom the likelihood of a natural conception was already significantly reduced^[11]^. The results clearly showed that the greatest contribution of ART to fertility growth related to older women, for whom time is limited in terms of the chances of having a natural birth.

## Data Availability

CZSO 2023. Population changes—time series. Available at: https://www.czso.cz/csu/czso/population-changes-time-series [data downloaded 5.1.2023]. IHIS 2022. Anonymised linked individual data on live births from the vital statistics (the Czech Statistical Office) and the National Register of Assisted Reproduction (IHIS)—unpublished data prepared under license for the scientific purposes of Department of Demography and Geodemography, Charles University. The data that support the findings of this study are available from the Institute of Health Information and Statistics of the Czech Republic but restrictions apply to the availability of these data, which were used under license for the current study, and so are not publicly available. Data are however available from the authors upon reasonable request and with permission of the Institute of Health Information and Statistics of the Czech Republic.
